# The Role of Biomarkers in Personalized Anesthesia: From Physiological Parameters to Molecular Diagnostics

**DOI:** 10.3390/biomedicines14020300

**Published:** 2026-01-29

**Authors:** Irina Nenadic, Predrag Stevanovic, Marina Bobos, Maja Stojanovic, Nemanja Dimic, Suzana Bojic, Dragica Dekic, Jovana Radovanovic, Marko Djuric

**Affiliations:** 1 Clinic for Anesthesiology and Intensive Care, University Clinical Hospital Center “Dr Dragisa Misovic—Dedinje”, 11000 Belgrade, Serbia; 2 Department of Anesthesiology, Reanimatology and Intensive Care, Faculty of Medicine, University of Belgrade, 11000 Belgrade, Serbia; 3 The Institute for Cardiovascular Diseases “Dedinje”, 11000 Belgrade, Serbia; 4 Department of Physiology, Faculty of Medical Sciences, University of Kragujevac, 34000 Kragujevac, Serbia

**Keywords:** biomarkers, personalized anesthesia, inflammation, oxidative stress, neurotoxicity, acute kidney injury, pharmacogenomics, microRNAs, digital biomarkers, perioperative monitoring

## Abstract

Personalized anesthesia has emerged as a key direction in modern perioperative medicine, driven by advances in molecular biology, analytical technologies, and digital monitoring. Traditional physiological parameters often fail to detect early stages of organ dysfunction, whereas molecular biomarkers provide earlier and more sensitive insight into inflammation, oxidative stress, neurotoxicity, and renal or hepatic injury. Inflammatory markers such as IL-6, CRP, and PCT indicate early immune activation, while oxidative stress biomarkers, including 8-isoprostanes and malondialdehyde, quantify metabolic imbalance and ischemia–reperfusion injury. Neurotoxicity biomarkers such as S100β, NSE, and GFAP allow early detection of subclinical cerebral injury, whereas kynurenine-pathway metabolites reflect neuroinflammation and the risk of postoperative cognitive dysfunction. Renal biomarkers such as NGAL, KIM-1, and cystatin C detect acute kidney injury significantly earlier than creatinine, and miR-122 holds strong potential as an early marker of hepatocellular injury. Genetic and epigenetic biomarkers—including polymorphisms in CYP2D6, CYP3A4/5, RYR1, OPRM1, and COMT, as well as microRNA-based signatures—enable individualized drug dosing and optimization of anesthetic strategies. Meanwhile, digital biomarkers such as EEG-derived indices, HRV, and NIRS provide continuous real-time physiological monitoring and can integrate with AI-based algorithms for predictive, adaptive anesthesia management. Although no single biomarker meets all criteria for an ideal clinical indicator, combining molecular, genetic, and digital biomarkers represents the most promising pathway toward fully personalized, safe, and outcome-optimized perioperative care.

## 1. Introduction

In recent decades, anesthesiology has shifted from standardized anesthetic techniques and weight-based drug dosing toward individualized anesthesia, in which drug selection, dosing, and anesthetic strategies are tailored to the individual patient. Traditional clinical and hemodynamic parameters, although indispensable, often lack sufficient sensitivity to detect early organ dysfunction, inflammation, or ischemic injury. This limitation has stimulated growing interest in biomarkers as tools for more precise perioperative assessment.

According to the National Institutes of Health (NIH), a biomarker is a “characteristic that is objectively measured and evaluated as an indicator of normal biological processes, pathogenic processes, or pharmacologic responses to a therapeutic intervention” [1]. In practice, biomarkers include molecules, proteins, genes, physiological signals, and digital parameters that reflect specific biological states. In anesthesiology, they bridge the gap between conventional monitoring and personalized medicine, allowing refined risk stratification, optimized anesthetic protocols, and early detection of complications. Inflammatory biomarkers such as interleukin-6 (IL-6), C-reactive protein (CRP), and procalcitonin predict postoperative morbidity. IL-6 is a proinflammatory cytokine that rises rapidly in response to surgical stress, serving as an early indicator of immune activation. Systematic reviews have confirmed associations between elevated IL-6 and increased postoperative morbidity and mortality in non-cardiac surgery [2,3]. Renal injury markers, such as neutrophil gelatinase-associated lipocalin (NGAL) and kidney injury molecule-1 (KIM-1), enable detection of kidney injury before serum creatinine becomes abnormal [4].

Despite extensive research, routine clinical implementation of biomarkers in anesthesiology remains limited. Barriers include insufficient methodological standardization, high analytical costs, interindividual and population heterogeneity, and the need for validation in large, multicenter clinical trials. These limitations currently restrict clinicians from systematically using many biomarkers for perioperative risk assessment or therapy adjustments. In parallel, the emergence of digital biomarkers and artificial intelligence-based analytical approaches has expanded perioperative monitoring beyond laboratory measurements, introducing new technical, ethical, and interpretative challenges that may affect clinical decision-making and integration into daily anesthetic practice [5].

To guide the reader from background concepts to detailed evidence, this narrative review summarizes current evidence on biomarkers relevant to anesthesia and perioperative medicine. It emphasizes their functional classification, biological mechanisms, clinical applicability, limitations, and future perspectives. Particular focus is placed on integrating biomarkers into personalized anesthetic strategies, a key direction in the evolution of modern anesthesiology.

From a clinical and translational perspective, biomarkers used in anesthesia and perioperative medicine can be further classified not only by biological domain but also by intended clinical function and level of validation. This distinction is essential to avoid overinterpretation of emerging biomarkers and to guide appropriate clinical implementation.

1. Diagnostic biomarkers are primarily used to detect disease or subclinical organ dysfunction. In perioperative practice, well-established and validated diagnostic biomarkers include cardiac troponins or N-terminal pro-Brain Natriuretic Peptide (NT-proBNP) for myocardial injury, serum creatinine for acute kidney injury, and lactate as an indicator of global tissue hypoperfusion [6]. These biomarkers are supported by guideline recommendations and are routinely implemented in clinical workflows.
2. Predictive biomarkers estimate the likelihood of future complications or differential response to interventions. Inflammatory mediators such as IL-6, IL-10, and procalcitonin have been investigated as predictors of postoperative complications, including infection, delirium, and prolonged recovery [7].
3. Prognostic biomarkers provide information on disease severity or long-term outcomes. Examples include NT-proBNP for predicting postoperative cardiovascular risk, glial fibrillary acidic protein (GFAP) for neurological injury and cognitive outcomes, and NGAL and cystatin-C for renal injury [6,7,8]. While some prognostic biomarkers, such as NT-proBNP and troponin, are well validated and incorporated into perioperative risk stratification strategies, others, like GFAP, are supported by growing clinical evidence but are not yet universally standardized.
4. Monitoring biomarkers shows real-time changes in the body in response to anesthesia and surgery. These include cortisol, glucose, and lactate [9]. Recent focus is on digital biomarkers, such as heart rate changes, brain activity patterns, and tissue oxygen levels measured by near-infrared light. These give real-time insight into stress, anesthesia depth, and blood flow. Using them with molecular markers and artificial intelligence may support personalized anesthesia [10]. Some of these markers are widely used, while others are still being studied and lack clear guidelines.

**Figure 1:**
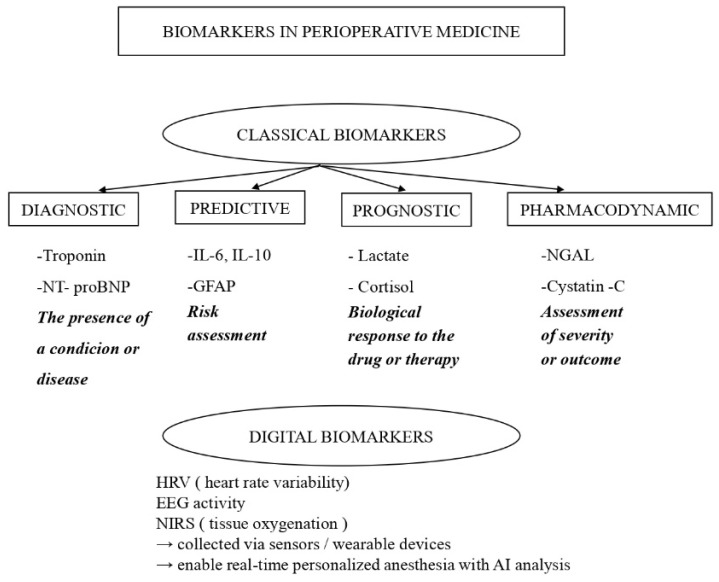
Classification of Biomarkers.

The functional classification of perioperative biomarkers into diagnostic, predictive, prognostic, pharmacodynamic, and digital categories is summarized in Figure 1.

Importantly, biomarkers differ in their stage of clinical validation. Conventional lab markers and digital measures are generally validated and widely used, while new biomarkers—such as microRNAs, epigenetic markers, and combined panels—remain mostly experimental. These newer biomarkers may enable earlier detection of organ injury and improved risk assessment, but currently lack standardized tests, reference values, and broad validation.

Recognizing both the functional role and validation status of biomarkers is crucial for their responsible integration into personalized anesthesia. Although no single biomarker fulfills all criteria of an ideal clinical indicator, combined biomarker approaches represent the most promising pathway toward precision perioperative care [11]. In clinical practice, most emerging biomarkers should be used to support existing parameters, potentially improving perioperative decision-making by adding nuanced insights to conventional monitoring and clinical judgment. 

## 2. Methods

This article was conducted as a structured narrative review, informed by the scope, design, and methodological characteristics of the studies included in the final reference list (*n* = 81). The cited literature encompasses systematic reviews and meta-analyses, prospective and retrospective observational studies, randomized and non-randomized clinical trials, pharmacogenetic association studies, and digital monitoring research relevant to anesthesia and perioperative medicine.

Literature sources were identified through structured searches of PubMed/MEDLINE, Scopus, Web of Science, and Google Scholar, focusing on human studies relevant to anesthesia and perioperative medicine. Searches combined Medical Subject Headings (MeSH) and free-text terms for anesthesia and perioperative care, including inflammation biomarkers and anesthesia, oxidative stress biomarkers and anesthesia, genetic biomarkers and anesthesia, epigenetic biomarkers and anesthesia, metabolic biomarkers and anesthesia, digital biomarkers and anesthesia, renal biomarkers and anesthesia, and neuroprotection biomarkers and anesthesia.

The review emphasizes studies published in the past five years (through December 2025) and presents recent evidence on molecular, genetic, epigenetic, metabolic, and digital biomarkers, as well as emerging AI-based perioperative monitoring and decision-support applications. Only select seminal studies were included to contextualize foundational biomarker definitions, pharmacogenetic mechanisms, and established perioperative monitoring principles.

**Figure 2:**
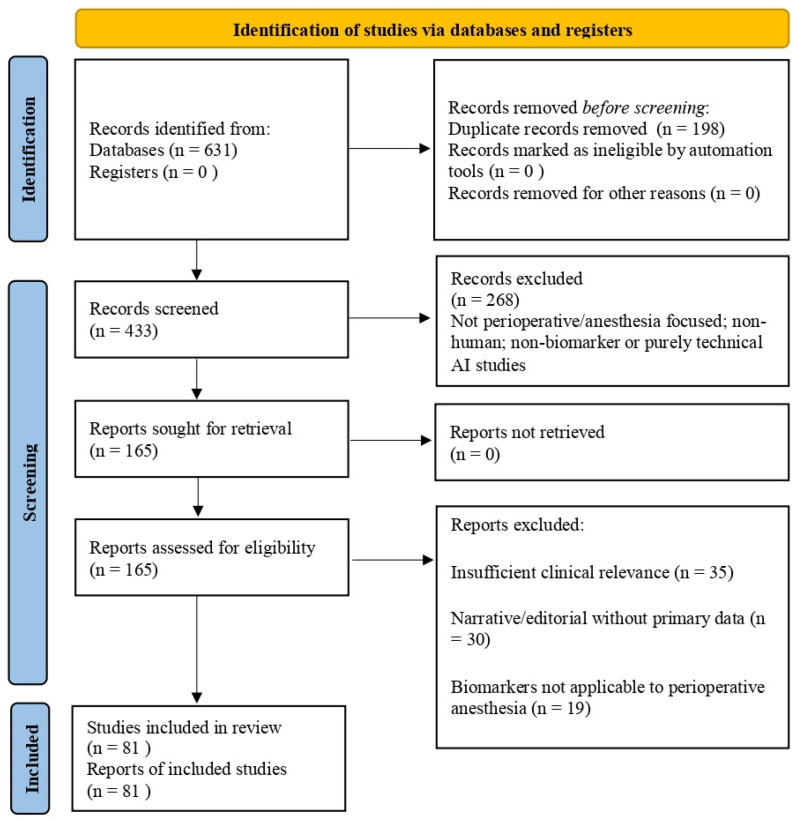
PRISMA flow diagram.

Study selection was guided by relevance to anesthesia or perioperative care, clinical or translational applicability, and inclusion of patient-based data or validated systematic syntheses. Editorials, narrative opinions without primary data, and non-translational animal studies were excluded. The study identification and selection process underlying the final reference list is summarized in a PRISMA-inspired flow diagram (Figure 2).

Given the heterogeneity of biomarker categories, study designs, patient populations, and outcome measures, a formal meta-analysis was not performed. Methodological quality was assessed qualitatively, with particular attention to study design, sample size, outcome definition, and level of clinical validation.

## 3. Biomarkers Relevant to Anesthesia 

(a) Biomarkers of Stress and Inflammation.

The perioperative period represents a major physiological challenge, as surgical trauma and anesthesia activate neuroendocrine and immune pathways. These responses are reflected by changes in stress and inflammatory biomarkers, whose dynamics correlate with perioperative burden and the risk of complications.

Interleukin-6 (IL-6) is a key proinflammatory cytokine that rises rapidly in response to surgical stress and serves as an early indicator of immune activation. IL-6 levels correlate with procedural invasiveness and are associated with postoperative complications [12]. In contrast, C-reactive protein (CRP) reflects the later phase of systemic inflammation, with concentrations increasing within hours and peaking several days postoperatively, thereby complementing IL-6 in perioperative assessment [13].

Procalcitonin (PCT), although primarily a marker of bacterial infection, may also increase after major surgery. Its perioperative utility lies in differentiating sterile inflammatory responses from infectious complications and enabling early detection of postoperative sepsis [14].

Cortisol remains a classical marker of neuroendocrine stress, increasing rapidly during surgery and reflecting the magnitude of the stress response. Its perioperative dynamics may indicate how different anesthetic techniques modulate neuroendocrine activation [15].

High-mobility group box 1 protein (HMGB1) has emerged as an important marker of tissue injury and innate immune activation. Released from damaged cells, HMGB1 acts as an alarmin and contributes to systemic inflammation. Elevated perioperative HMGB1 levels correlate with surgical trauma severity and have been implicated in sepsis-like complications; recent randomized data demonstrate significant postoperative increases following major abdominal surgery (HMGB1 levels were significantly elevated at the end of colorectal surgery, ~235% above preoperative values, and on the first postoperative day, ~90% increase) [16].

(b) Biomarkers of Oxidative Stress and Ischemia–Reperfusion Injury.

Oxidative stress results from an imbalance between the production of reactive oxygen and nitrogen species (ROS/RNS) and antioxidant defenses and plays a central role in perioperative tissue injury, ischemia–reperfusion damage, and systemic inflammation [17,18]. Consequently, oxidative stress biomarkers have gained attention as indicators of cellular injury and predictors of postoperative outcomes [19,20].

Lactate is widely used as an indirect marker of oxidative stress and impaired mitochondrial function. Elevated perioperative lactate levels are associated with increased risk of complications, prolonged intensive care stay, and mortality, while lactate clearance has prognostic value for early postoperative outcomes. The prognostic value of perioperative lactate has been supported by observational and multicenter cohort studies in cardiovascular and major vascular surgery, demonstrating associations between elevated lactate levels, impaired microcirculatory flow, and adverse postoperative outcomes [21,22].

Among direct oxidative stress markers, 8-isoprostanes (8-iso-PGF_2_α) are considered the gold standard for assessing lipid peroxidation. Elevated levels have been reported after cardiovascular, abdominal, and transplant surgery and correlate with operative duration and reperfusion stress [23,24]. Malondialdehyde (MDA), another lipid peroxidation product, also increases perioperatively and reflects the intensity of metabolic and inflammatory stress, with declining levels indicating recovery and antioxidant adaptation [18,25].

Total antioxidant capacity (TAC) integrates enzymatic (superoxide dismutase—SOD, glutathione peroxidase—GPx, catalase—CAT) and nonenzymatic antioxidant defenses such as glutathione and vitamins C and E. Reduced TAC and decreased antioxidant enzyme activity are commonly observed during and after major surgery and are associated with higher complication rates and delayed recovery [18,25].

Anesthetic agents may influence oxidative balance: propofol exhibits antioxidant properties and may preserve TAC, whereas volatile anesthetics show variable effects, potentially inducing mild oxidative stress while also exerting protective preconditioning through adaptive responses [26].

(c) Biomarkers of Neuroprotection and Neurotoxicity.

Perioperative neurological injury remains a major concern, particularly during cardiac and neurosurgical procedures, where hypoperfusion, microembolization, hypoxia, and neuroinflammation may lead to postoperative delirium or cognitive dysfunction [27]. Biomarkers of neural injury are increasingly used for early detection and monitoring of these processes.

S100β, neuron-specific enolase (NSE), and glial fibrillary acidic protein (GFAP) are the most extensively studied biomarkers of neurotoxicity. S100β, released from astrocytes following blood–brain barrier disruption, is a sensitive early marker of cerebral injury [27]. A recent meta-analysis including 30 studies demonstrated that postoperative elevations of S100β and neuron-specific enolase were significantly associated with postoperative cognitive dysfunction [28]. NSE reflects direct neuronal damage and complements S100β by indicating neuronal necrosis or apoptosis; elevated postoperative NSE levels are associated with cognitive impairment, particularly in older patients [29]. 

GFAP, a structural astrocytic protein, reflects astroglial activation and grey matter injury and has emerged as a reliable marker of ischemic and traumatic brain injury as well as perioperative neuroinflammation [30,31]. In a prospective observational study including 58 patients, perioperative GFAP dynamics reflected early brain injury and subsequent cognitive changes after deep-brain stimulation surgery [30]. Combined assessment of S100β, NSE, and GFAP provides a biomarker panel for early detection of subclinical cerebral injury and evaluation of neuroprotective anesthetic strategies.

The kynurenine pathway further links inflammation with neurotoxicity. Activation of indoleamine-2,3-dioxygenase shifts tryptophan metabolism toward kynurenine production during inflammation. Elevated kynurenine levels and increased KYN/TRP ratios have been associated with postoperative cognitive impairment and delirium risk, while experimental inhibition of this pathway mitigates cognitive deficits [32,33,34]. Importantly, the balance between neurotoxic metabolites (e.g., quinolinic acid) and neuroprotective kynurenic acid, expressed as the KYNA/QUIN ratio, reflects the equilibrium between neurotoxicity and neuroprotection [35].

Integration of neuroinjury biomarkers with digital monitoring tools, such as EEG-derived indices and hemodynamic parameters, represents an important step toward individualized, neuroprotective anesthesia, particularly in high-risk patient populations.

(d) Biomarkers of Renal and Hepatic Function.

Conventional diagnostic criteria for acute kidney injury (AKI) rely on increases in serum creatinine and/or reduced urine output; however, creatinine is a late functional marker that often rises only after substantial renal damage has occurred. Consequently, recent research has focused on biomarkers of early tubular injury that reflect structural kidney damage rather than delayed functional impairment. Among these, neutrophil gelatinase-associated lipocalin (NGAL), kidney injury molecule-1 (KIM-1), and cystatin C are the most extensively studied [36].

Clinical evidence supports the early diagnostic value of these biomarkers in the perioperative setting. In a recent prospective study involving 488 patients undergoing major abdominal surgery, urinary NGAL, KIM-1, dickkopf-related protein 3 (DKK-3), and the product of insulin-like growth factor binding protein-7 (IGFBP-7) and tissue inhibitor of metalloproteinases-2 (TIMP-2) were measured shortly after surgery. NGAL was the best predictor of postoperative AKI [37]. Subsequent studies in cardiac surgery and critically ill ICU populations confirmed that NGAL and KIM-1 levels increase within 4–12 h after surgical stress, significantly earlier than creatinine, while cystatin C correlates more closely with glomerular filtration and provides prognostic value for early AKI detection [38,39,40].

Nevertheless, no single biomarker provides perfect discrimination. In a mixed ICU population, NGAL and cystatin C showed considerable overlap between AKI and non-AKI patients, limiting their standalone predictive accuracy [41]. Current evidence therefore supports a multimarker approach, integrating renal biomarkers with clinical assessment to identify subclinical tubular injury and guide early nephroprotective strategies.

Traditional markers of hepatic injury, including aspartate and alanine aminotransferases (AST and ALT), remain part of routine perioperative assessment but are limited by delayed elevation and low specificity, as increases may occur secondary to muscle injury, hemolysis, or systemic stress [42]. This has led to growing interest in circulating microRNAs, particularly miR-122, which constitutes the majority of hepatocellular microRNA content and is rapidly released into circulation following hepatocyte injury [43].

Recent studies demonstrate that miR-122 is a more sensitive and specific early marker of hepatocellular injury than AST and ALT, with earlier elevations during ischemia–reperfusion injury, laparoscopic procedures, major hepatic resections, and transplantation [44,45]. Biomarker panels combining miR-122 with classical enzymes and functional liver parameters improve the prediction of post-hepatectomy liver failure and early detection of drug- or ischemia-induced liver injury. Although clinical implementation requires further standardization, current data indicate that miR-122 is the most promising early hepatic biomarker in perioperative medicine.

During major surgical procedures—particularly cardiac, transplant, abdominal, and vascular surgery—the kidneys and liver are frequently exposed to hypotension, hypoperfusion, inflammation, drug toxicity, and ischemia–reperfusion injury. In this context, early and sensitive biomarkers provide clinically valuable information long before conventional markers become abnormal.

(e) Genetic and Epigenetic Biomarkers in Anesthesia.

Genetic biomarkers encompass inherited gene variants that influence anesthetic drug metabolism, efficacy, and susceptibility to adverse reactions, forming a cornerstone of personalized anesthesia.

Classic examples include butyrylcholinesterase (BCHE) deficiency, identified in patients experiencing prolonged neuromuscular blockade after succinylcholine administration [46], and malignant hyperthermia, a life-threatening pharmacogenetic disorder associated with autosomal dominant mutations in the ryanodine receptor gene (RYR1) [47,48]. These discoveries laid the foundation for modern pharmacogenetics in anesthesiology.

Interindividual variability in opioid response is partly explained by polymorphisms in the OPRM1 gene, particularly the A118G variant, which alters μ-opioid receptor function and influences analgesic efficacy across populations [49]. Similarly, polymorphisms in the catechol-O-methyltransferase (COMT) gene affect catecholamine metabolism and pain modulation. The Val158Met variant has been associated with differences in opioid requirements and pain sensitivity, particularly in cancer pain management. Associations between OPRM1 and COMT polymorphisms and opioid analgesic response have been demonstrated in multiple pharmacogenetic association studies across different surgical and pain populations, indicating clinically relevant interindividual variability in analgesic requirements [50,51,52,53,54].

Cytochrome P450 enzymes represent the most clinically relevant pharmacogenetic system in anesthesia. CYP2D6 polymorphisms influence the metabolism of opioids such as tramadol and codeine, resulting in either reduced analgesia or increased toxicity [55,56]. Variants in CYP2C9 and CYP2C8 affect NSAID pharmacokinetics, while CYP2C19 and CYP2B6 polymorphisms alter benzodiazepine metabolism and safety profiles [56,57]. CYP2B6 also plays a dominant role in propofol metabolism, contributing to variability in anesthetic depth and recovery [58].

The CYP3A4/5 enzyme system metabolizes approximately half of the commonly used anesthetic drugs, including fentanyl, midazolam, and lidocaine [59]. Genetic variants, such as CYP3A4*20 and CYP3A5 loss-of-function alleles, significantly influence drug clearance and plasma concentrations, with notable ethnic variability [60,61,62].

Postoperative cognitive dysfunction (POCD) is a frequent complication after anesthesia and surgery in elderly patients, severely affecting recovery and quality of life. Although its mechanisms are not fully understood, studies suggest that the interaction between silent mating type information regulation two homolog 1 (SIRT1) and brain-derived neurotrophic factor (BDNF) is a key regulator of cognition and neurodegeneration. Studies in aged mice suggest that reduced SIRT1/BDNF levels, leading to altered synaptic plasticity and neuronal excitability, may contribute to postoperative cognitive decline [63].

The GABRA1 rs2279020 polymorphism influences response to propofol, with GG carriers demonstrating faster, more efficient sedation and less hypotension than AG/AA carriers. This genetic variation may aid in individualized dosing and reducing adverse effects during anesthesia induction [64].

The GABRA1 rs4263535 variant is associated with more profound and faster sedation with midazolam; carriers of the minor (A) allele reach desired sedation levels more quickly, highlighting its role in optimizing benzodiazepine dosing and safety [65].

Beyond inherited polymorphisms, epigenetic mechanisms—including DNA methylation, histone modifications, and microRNA regulation—modulate gene expression in response to environmental factors, medications, and physiological stress [66]. Epigenetic alterations in genes encoding inflammatory mediators and drug-metabolizing enzymes have been linked to postoperative delirium, neurocognitive dysfunction, and altered analgesic response [67].

MicroRNAs such as miR-21, miR-155, miR-210, miR-146a, and miR-451a reflect systemic inflammation, hypoxia, and opioid responsiveness, while miR-122 links epigenetic regulation with hepatic injury [68]. Experimental data indicate that anesthetics can modify histone acetylation in brain regions involved in learning and memory, potentially contributing to postoperative cognitive dysfunction; these effects may be attenuated by histone deacetylase inhibition [69].

Although epigenetic and microRNA biomarkers offer valuable mechanistic insight into perioperative inflammation, neurotoxicity, and organ injury, their current role in clinical anesthesia remains exploratory. Most available evidence is derived from small observational cohorts or preclinical models, with substantial heterogeneity in sampling protocols and analytical pipelines; therefore, broader clinical implementation awaits cost reduction and formal integration into clinical guidelines. At present, these biomarkers should be viewed primarily as research tools rather than clinically deployable assays. 

(f) Digital Biomarkers and Biomarkers of Tissue Perfusion.

Digital biomarkers represent continuously measured physiological and neurophysiological parameters acquired via sensors, monitors, and algorithm-based analysis, enabling real-time, personalized perioperative management. Electroencephalographic indices, including bispectral index (BIS), entropy, and patient state index (PSI), are widely used to monitor anesthetic depth and reduce the risk of intraoperative awareness, excessive sedation, postoperative delirium, and cognitive dysfunction [70]. In a randomized controlled trial involving 180 pediatric surgical patients, BIS-guided titration of sevoflurane reduced anesthetic exposure without compromising hemodynamic stability [71].

Near-infrared spectroscopy (NIRS) provides noninvasive monitoring of cerebral and peripheral tissue oxygenation and is particularly valuable during cardiac and major vascular surgery or in states of potential hypoperfusion. This was documented in an observational study including 101 adult patients undergoing cardiac surgery with cardiopulmonary bypass [72].

Heart rate variability reflects the balance of the autonomic nervous system and correlates with anesthetic depth and stress responses. Intraoperative HRV has been proposed as a reliable indicator of autonomic homeostasis during anesthesia [73]. Digital monitoring also includes continuous hemodynamic parameters and metabolic indicators, such as lactate, which reflect tissue perfusion and oxygen utilization.

Artificial intelligence (AI) and machine learning increasingly enable the integration of digital biomarkers (e.g., EEG-derived indices, heart rate variability, near-infrared spectroscopy, hemodynamic waveforms) with laboratory-based biomarkers to support personalized perioperative care. Anesthesia is particularly well-suited to AI-based approaches, given the availability of continuous, high-frequency physiological data alongside intermittent molecular measurements [4].

Recent work highlights the potential of AI to enhance perioperative monitoring, particularly through advanced EEG signal analysis for anesthetic depth assessment using convolutional neural networks and hybrid deep learning models. In one narrative review, it was concluded that artificial intelligence has significant potential to improve EEG-based monitoring during anesthesia, but its clinical implementation is currently limited by insufficient generalizability, inadequate model validation, and challenges with data availability and interoperability [74]. A systematic review of 13 randomized controlled trials of machine learning–augmented interventions in perioperative settings demonstrates that AI-driven applications, such as hypotension and nociception indices, can reduce the duration of intraoperative hypotension and improve postoperative pain outcomes, albeit with variable effects on broader clinical endpoints [75].

Clinical explorations also suggest that AI could be useful for multimodal risk stratification when physiologic signals are integrated with biochemical markers, thereby supporting earlier detection of complications and optimizing anesthetic management. For example, deep learning models trained on high-dimensional perioperative data have shown promise for classifying anesthesia states and predicting adverse events, although these findings require broader validation [4,74,75].

Despite these advances, most AI-driven perioperative tools remain investigational. Key limitations include a lack of standardized input variables, limited external validation, potential bias in the training datasets, and challenges with interpretability and clinical workflow integration. At present, AI systems should be viewed as decision-support tools, complementing rather than replacing clinical judgment [74]. Large, prospective, multicenter validation studies will be essential to determine their clinical impact and cost-effectiveness.

Biomarkers of metabolic and cardiac stress, including lactate and cardiac troponin, provide early insight into tissue hypoxia and myocardial injury. Elevated perioperative lactate levels are associated with adverse outcomes, while dynamic changes reflect tissue perfusion status [76]. Postoperative troponin elevation, even in the absence of ischemic symptoms, identifies myocardial injury after non-cardiac surgery and is associated with increased short- and long-term mortality, supporting routine monitoring in high-risk patients [77].

Integrating digital biomarkers such as EEG indices, NIRS, HRV, lactate, and troponin with conventional clinical parameters enables a multidimensional assessment of microcirculatory adequacy, particularly in cases where macrocirculatory variables appear normal [78]. This multimodal approach supports timely intervention, optimization of anesthetic and analgesic dosing, and personalized neuro- and cardioprotection, advancing perioperative care toward predictive, preventive, and precision anesthesiology.

An overview of key biomarkers relevant to anesthesia and perioperative medicine, categorized by biological domain and clinical function, is summarized in Table 1.

## 4. Discussion

Biomarkers are increasingly central to personalized anesthesia, driven by advances in molecular diagnostics, analytical platforms, and digital monitoring. Compared with traditional perioperative parameters (blood pressure, heart rate, urine output, oxygen saturation), biomarkers may detect evolving organ injury and dysregulated physiology earlier, enabling proactive risk stratification, timely intervention, and individualized anesthetic strategies. For example, renal biomarkers such as NGAL and KIM-1 can identify acute kidney injury hours to days before creatinine rises, while inflammatory mediators such as IL-6 reflect early and intense immune activation after surgical stress.

Oxidative stress biomarkers (e.g., 8-isoprostanes, malondialdehyde) provide complementary prognostic information by quantifying lipid peroxidation and metabolic stress during ischemia–reperfusion and major surgery. When interpreted alongside antioxidant defenses (total antioxidant capacity, SOD, GPx, catalase), they help characterize the balance between oxidative injury and adaptive response. This may be clinically relevant for tailoring perioperative strategies, including selection of anesthetic regimens with potentially more favorable redox profiles, and for identifying patients at heightened risk for complications.

In the neurocognitive domain, biomarkers such as S100β, NSE, and GFAP have become important tools for detecting subclinical cerebral injury and neuroinflammation, particularly during cardiac and neurosurgical procedures. Their perioperative elevations are consistent with mechanisms such as hypoperfusion, microembolization, and inflammatory activation and may inform neuroprotective strategies. Additional mechanistic insight may be gained from kynurenine pathway profiling, which links systemic inflammation with neurochemical imbalance and may help identify patients at risk for delirium and postoperative cognitive dysfunction.

Pharmacogenetic biomarkers represent another key pillar of precision anesthesia. Polymorphisms in genes involved in drug metabolism and receptor sensitivity (e.g., CYP2D6, CYP2C19, CYP3A4/5, OPRM1, and COMT) as well as susceptibility genes for adverse reactions (e.g., RYR1 in malignant hyperthermia) can influence anesthetic efficacy, safety, and dosing requirements. However, broad implementation remains limited by cost, laboratory capacity, and the lack of standardized perioperative recommendations across healthcare systems.

**Figure 3:**
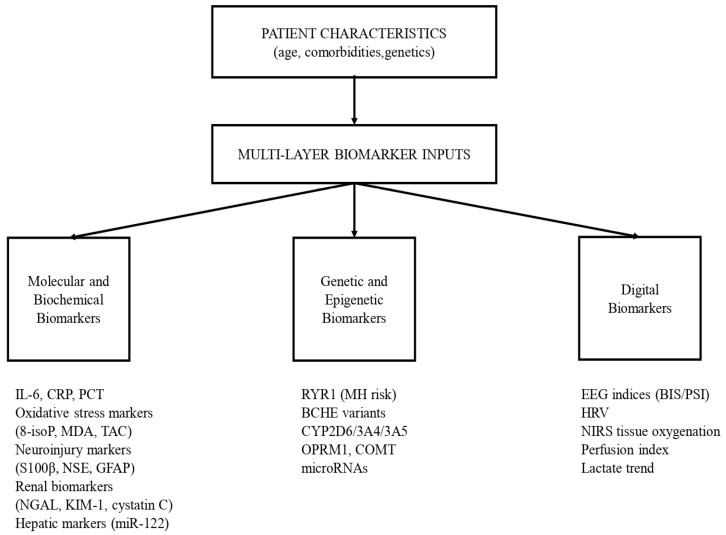
Real-Time Physiological Signals and Digital Biomarkers in Personalized Anesthesia.

Digital biomarkers bridge molecular and physiological monitoring by providing continuous, real-time insight into anesthetic depth, autonomic regulation, and tissue perfusion (e.g., EEG-derived indices, HRV, NIRS), and they can be integrated with AI-based analytics for predictive decision support. Yet challenges persist, including technical standardization, artifact management, and the need for clinician training to reliably interpret complex outputs. An integrative, multi-layer biomarker framework for personalized anesthesia is shown in Figure 3.

For emerging biomarker classes—especially epigenetic signatures and circulating microRNAs—evidence remains largely preclinical or heterogeneous. Although anesthetic exposure can alter microRNA expression linked to inflammation, oxidative stress, and apoptosis [79], robust human validation linking circulating microRNAs to clinically meaningful perioperative outcomes is still insufficient. Systematic reviews highlight substantial heterogeneity in candidate microRNAs for AKI prediction and lack of consistent replication across studies [80]. Reviews in anesthesiology similarly emphasize barriers such as inconsistent sampling and analytical pipelines, inter-institutional variability, and limited translational reproducibility. Therefore, while molecular and epigenetic biomarkers remain promising, their routine perioperative use requires standardized protocols, reference ranges, and prospective multicenter validation. 

Direct head-to-head comparisons between perioperative biomarkers remain limited, and clinically actionable sensitivity, specificity, or cutoff thresholds are not uniformly established. For many biomarkers, reported values vary substantially depending on the surgical population, sampling time, and analytical platform. Consequently, most biomarkers currently serve as risk-stratification tools rather than standalone diagnostic tests. Where available, threshold-based interpretation (e.g., postoperative troponin elevation, lactate clearance, NGAL fold-change) should be applied in conjunction with clinical context rather than as absolute decision triggers.

Beyond diagnostic performance, feasibility and cost-effectiveness are major determinants of biomarker adoption in routine anesthesia practice. Widely used biomarkers, including lactate, creatinine-adjacent renal markers, troponin, and standard inflammatory markers (e.g., C-reactive protein, procalcitonin), are inexpensive, rapidly available, and supported by existing hospital laboratory infrastructure, making them readily implementable in perioperative workflows In contrast, advanced molecular and epigenetic biomarkers, such as microRNA profiling and DNA methylation analyses, are associated with higher costs, longer turnaround times, and the need for specialized laboratory facilities, limiting their current use to research or selected tertiary centers. Digital biomarkers and AI-supported monitoring may reduce reliance on laboratory testing but require investment in monitoring hardware, data integration platforms, and clinician training. Overall, the feasibility of biomarker-guided personalized anesthesia depends not only on clinical validity but also on economic sustainability, infrastructure availability, and integration into existing perioperative systems.

Digital biomarkers may reduce laboratory costs but require investment in monitoring hardware, software integration, and clinician training. Comprehensive cost-effectiveness analyses comparing biomarker-guided strategies with standard care are scarce and represent a critical gap in the current evidence base.

Biomarker performance varies substantially across patient populations due to age, ethnicity, genetic background, and comorbidities. Older patients often exhibit altered baseline biomarker profiles and reduced organ reserve, which can affect the sensitivity and specificity of perioperative injury detection. Ethnic and genetic differences influence pharmacogenetic pathways and inflammatory responses, limiting the generalizability of biomarker thresholds derived from homogeneous cohorts [81]. Comorbid conditions such as chronic kidney disease, diabetes, and cardiovascular disease further modify biomarker kinetics and baseline levels. These factors challenge the use of universal cutoffs and emphasize the need for population-specific reference ranges and individualized interpretation within perioperative care.

**Figure 4:**
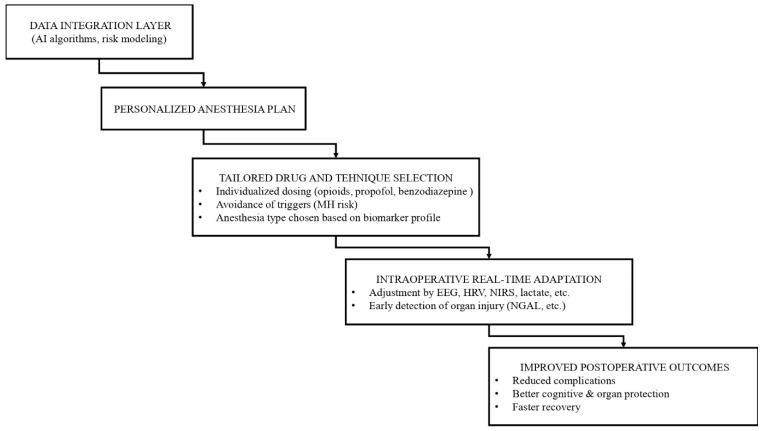
Conceptual Framework of Biomarker-Guided Personalized Anesthesia.

A practical workflow illustrating how integrated biomarker data and AI-based risk modeling can inform personalized anesthesia planning, intraoperative adaptation, and postoperative outcomes is presented in Figure 4.

**Figure 5:**
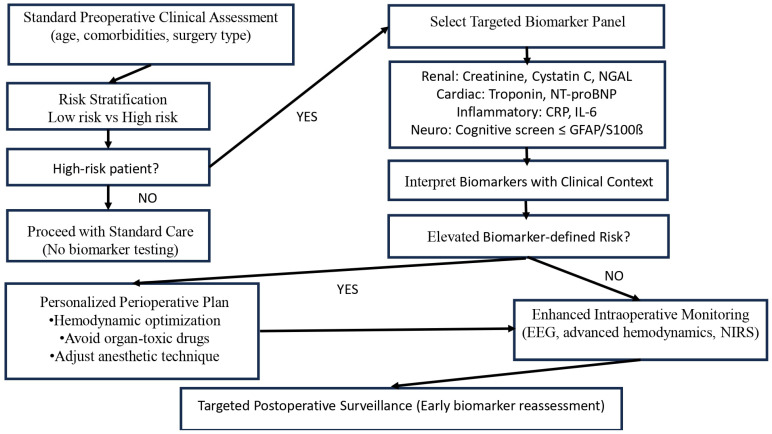
Biomarker—Guided Preoperative Flowchart (This algorithm represents a conceptual decision-support framework. Biomarker thresholds and interventions should be adapted to local protocols and patient-specific factors).

Clinical implementation of biomarkers should follow scenario-based integration within existing perioperative pathways. Biomarkers can support early risk stratification and guide preventive strategies, but should complement—not replace—clinical assessment. Embedding biomarker measurements into established frameworks enables practical and safe application in routine anesthesia care. Figure 5 illustrates a practical decision-making algorithm demonstrating how selected biomarker panels can be integrated into existing perioperative workflows to support personalized anesthesia.

## 5. Conclusions

Biomarkers are key enablers of personalized anesthesia, offering earlier and more mechanistic insight into inflammation, oxidative stress, neurotoxicity, and organ injury than conventional monitoring alone. Digital biomarkers and AI-driven integration expand real-time perioperative assessment, while genetic and epigenetic biomarkers provide a framework for individualized drug selection and dosing. Despite progress, implementation is constrained by heterogeneity, limited standardization, and cost. The most realistic path forward is multimodal integration—combining biomarker panels with clinical assessment and digital monitoring to improve perioperative safety and outcomes.

### Future Directions

Future efforts should prioritize validating multimarker perioperative panels, developing standardized sampling and analytical pipelines, and integrating molecular biomarkers with digital signals (EEG, NIRS, HRV) to build multidimensional risk models. AI-enabled systems capable of real-time synthesis of hemodynamics, metabolic stress, and neuroinflammatory markers may support adaptive anesthesia strategies. Large prospective trials linking biomarker-guided interventions to patient-centered outcomes (organ function, delirium/POCD, recovery time, complications, mortality) will be essential to translate biomarker science into routine perioperative practice.

## Figures and Tables

**Table 1:** Biomarkers Relevant to Anesthesia.

Category	Biomarker	Function/Clinical Significance	Perioperative Dynamics/Notes
**Stress & Inflammation**	IL-6	Proinflammatory cytokine; early indicator of immune activation and acute inflammation	Rapid increase after surgical stress; correlates with surgery intensity and postoperative complications
	CRP	Reflects systemic inflammatory response	Increases 6–12 h after stimulus, peaks at 48–96 h; shows later systemic response
	Procalcitonin (PCT)	Marker of bacterial inflammation; differentiates sterile vs. infectious inflammation	May increase after major surgery; early detection of postoperative infections
	Cortisol	Neuroendocrine stress marker	Rapid rise during surgery; reflects stress intensity and anesthesia effect
	HMGB1	Nuclear protein and damage-associated molecular pattern (DAMP)	Reflects tissue injury; significantly elevated immediately after colorectal surgery and first postoperative day
**Oxidative Stress & Ischemia–Reperfusion**	Lactate	Indicator of oxidative stress and mitochondrial dysfunction	Elevated perioperatively; lactate clearance has prognostic value
	8-isoprostane (8-iso-PGF_2_α)	Lipid peroxidation product; “gold standard” for lipid oxidative damage	Elevated after cardiovascular, abdominal, and transplant surgeries; correlates with surgery duration and reperfusion stress
	Malondialdehyde (MDA)	Marker of lipid peroxidation and cellular membrane damage	Increases perioperatively; decrease during recovery reflects antioxidant adaptation
	Total Antioxidant Capacity (TAC)	Overall antioxidant defense	Includes enzymatic (SOD, GPx, CAT) and non-enzymatic components (glutathione, vitamins C and E); often decreases intra/postoperatively
**Neuroprotection & Neurotoxicity**	S100β	Calcium-binding protein; astrocyte marker	Early indicator of blood–brain barrier disruption; elevated after neuro or cardiac surgery
	NSE	Neuron-specific enzyme	Indicates neuronal necrosis or apoptosis; early postoperative rise correlates with cognitive deficits
	GFAP	Astrocytic structural protein	Marker of glial activation and ischemic/traumatic brain injury
	Kynurenine (KYN)	Metabolite of tryptophan pathway; immune-metabolic marker	Increase reflects IDO/TDO activity and systemic inflammation; imbalance (KYNA/QUIN) indicates neurotoxicity vs. neuroprotection
**Kidney & Liver Function**	NGAL	Marker of tubular injury	Rises 4–12 h post-surgery; early AKI detection
	KIM-1	Tubular injury marker	Increases early in AKI; complements NGAL
	Cystatin C	Glomerular filtration indicator	Early AKI detection; better correlation with GFR than creatinine
	AST/ALT	Traditional hepatic injury markers	Late rise; limited specificity
	miR-122	Liver-specific circulating microRNA	Early and sensitive marker of hepatocellular injury; superior to AST/ALT
**Genetic & Epigenetic**	BCHE	Butyrylcholinesterase deficiency	Causes prolonged paralysis after succinylcholine
	RYR1	Malignant hyperthermia susceptibility	Mutation causes malignant hyperthermia under anesthesia
	OPRM1 (A118G)	µ-opioid receptor	Alters opioid sensitivity; variable response to analgesics
	COMT (Val108/158Met)	Catecholamine metabolism	Polymorphism affects pain modulation and opioid requirements
	CYP2D6	Opioid metabolism	Ultra-rapid or poor metabolizer status affects drug efficacy/toxicity
	CYP2C9, CYP2C19, CYP2B6, CYP3A4/5	Metabolism of NSAIDs, benzodiazepines, propofol, opioids	Polymorphisms alter drug pharmacokinetics; may require dose adjustment
	SIRT1/BDNF	Neurocognitive regulation	Altered levels associated with postoperative cognitive dysfunction
	GABRA1 (rs2279020, rs4263535)	GABA receptor subunit	Influences sedative response to propofol/midazolam
	miR-21, miR-155, miR-210, miR-451a, miR-146a	Epigenetic regulation	Reflect systemic/neuroinflammation, hypoxia, opioid response
**Digital & Tissue Perfusion**	BIS, Entropy, PSI	EEG-based depth of anesthesia monitors	Prevents over- or under-sedation; reduces risk of intraoperative awareness and postoperative delirium
	NIRS	Tissue oxygenation	Noninvasive monitoring of brain/muscle perfusion; useful in high-risk surgeries
	HRV	Autonomic nervous system state	Correlates with anesthesia depth and hemodynamics
	Lactate, Troponin (cTn), Endothelin	Perfusion/metabolic stress markers	Elevated levels correlate with poor perioperative outcomes; combination with digital markers improves tissue perfusion assessment

## Data Availability

No new data were created or analyzed in this study.
